# GATA Binding Protein 3 Boosts Extracellular ATP Hydrolysis and Inhibits Metastasis of Breast Cancer by Up-regulating Ectonucleoside Triphosphate Diphosphohydrolase 3

**DOI:** 10.7150/ijbs.35563

**Published:** 2019-09-07

**Authors:** Meifang Li, Yuzhu Qi, Min Chen, Zun Wang, De Zeng, Yingsheng Xiao, Shaozhong Li, Haoyu Lin, Xiaolong Wei, Guojun Zhang

**Affiliations:** 1 ChangJiang Scholar's Laboratory of Shantou University Medical College, 22 Xinling Road, Shantou, China; 2 The Breast Center, Cancer Hospital of Shantou University Medical College, 7 Raoping Road, Shantou, China; 3 The Cancer Center and the Department of Breast and Thyroid Surgery, Xiang'an Hospital of Xiamen University, 2000 East Xiang'an Rd, Xiang'an, Xiamen, China; 4 Department of Medical Oncology, Cancer Hospital of Shantou University Medical College, 7 Raoping Road, Shantou, China; 5 Department of Thyroid Surgery, Central Hospital of Shantou, 114 Waima Road, Shantou, China; 6 Department of Breast and Thyroid Surgery, the First Affiliated Hospital of Shantou University Medical College, 57 Changping Road, Shantou, China; 7 Department of Pathology, Cancer Hospital of Shantou University Medical College, 7 Raoping Road, Shantou, China

**Keywords:** Breast Cancer, GATA3, ENTPD3, Extracellular ATP, Metastasis

## Abstract

Despite remarkable advancements in our understanding of breast cancer, it remains the leading cause of cancer deaths in women. Distant recurrence and metastasis is the main reason for death due to breast cancer. It is well recognized that the GATA binding protein 3 (GATA3), a transcription factor, is a tumor suppressor in breast cancer. To date, the mechanistic molecular details of GATA3 remain elusive, because, as a transcription factor, it is not a direct executor in physiological and pathological processes. Here, we demonstrate that GATA3 reduces the ATP level in the breast cancer microenvironment and inhibits breast cancer metastasis by up-regulating ectonucleoside triphosphate diphosphohydrolase 3 (ENTPD3). The extracellular ATP concentration is significantly higher in tumor tissues than in normal tissues and promotes the migration of cancer cells from the primary site. ENTPD3 hydrolyzes ATP in tumor microenvironment and suppresses breast cancer metastasis. Furthermore, ENTPD3 inhibits epithelial-to-mesenchymal transition, a key program responsible for the development of metastatic disease. These findings provide novel insights into the tumor suppressor activity of GATA3.

## Introduction

Breast cancer is the most common malignancy among women worldwide. Although the death rate from breast cancer has decreased by 39% in the past 30 years, it is still the primary cause of cancer deaths in women 1. The major reason for breast cancer-related deaths is distant metastasis. Approximately, 3-8% of new cases present with metastatic breast cancer and 20-30% of early stage cancers relapse with metastatic disease after comprehensive therapy 2-4. Therefore, a better understanding of the molecular mechanisms of metastasis is crucial to improving the outcome of patients with this type of cancer.

GATA binding protein 3 (GATA3), a transcription factor that regulates normal cell differentiation, serves as a biomarker of luminal epithelial cells and is involved in the progression of breast cancer 5-7. The expression of GATA3 is usually absent in triple negative breast cancer (TNBC) with a poor prognosis but is present in the luminal subtype with a favorable prognosis 8. Recent studies demonstrated that GATA3 suppressed breast cancer dissemination from the primary site by inhibiting epithelial-to-mesenchymal transition (EMT) and alterations of the tumor microenvironment, such as angiogenesis, collagen remodeling, and proteolysis 9-11. Though it is widely accepted that GATA3 is a tumor suppressor, the detailed mechanism repressing the progression of breast cancer is not entirely clear.

ATP is a well-established source of energy for living cells. Several decades back, Burnstock described that ATP was not only a source of energy but also a signaling molecule 12. Extracellular ATP (eATP) is a pivotal component of the extracellular matrix and has been shown to be involved in a variety of cellular processes 13. In normal tissues, eATP is present in minute amounts (nmol/L) 14, whereas its concentration rises up to several hundred μM or higher in cancer tissues 15, 16. Numerous studies have demonstrated that eATP promoted the metastasis of various cancers including breast, lung, liver, et.al 17-24. The primary mechanism of the pro-metastatic behavior of eATP is believed to involve boosting the concentration of intracellular free Ca^2+^ that triggers EMT and the release of several cellular factors 18, 23, 25. Thus, the eATP signaling pathway may be a potential anticancer target.

Ectonucleoside triphosphate diphosphohydrolase 3 (ENTPD3) is a member of the eATP hydrolytic enzyme family that converts eATP to ADP/AMP and modulates the eATP signaling pathway 26. ENTPD3 inhibited epithelial cell migration induced by eATP release after injury in larval zebrafish tail fins 27. In a mouse model of bladder cancer, the expression of ENTPD3 gradually decreased during cancer progression 28. Analysis of the gene expression omnibus (GEO) database (see below) showed that GATA3 up-regulated ENTPD3 in MDA-MB-231 breast cancer cells. Moreover, ENTPD3 was a biomarker of a favorable prognosis in patients with breast cancer derived from a Kaplan-Meier Plotter database (see below). Thus, these results suggest that ENTPD3 is a tumor suppressor in breast cancer. However, the significance of ENTPD3 in human cancers remains unclear. We hypothesize that GATA3 promotes the hydrolysis of ATP in the tumor microenvironment and inhibits the metastasis of breast cancer through a GATA3-ENTPD3-eATP axis. In the present study, we verify that ENTPD3 is a novel downstream target of GATA3 and is essential for its role as a tumor suppressor. Our findings provide a new perspective on the tumor suppressor activity of GATA3 in mammary epithelial cells.

## Materials and Methods

### Bioinformatics

Chu et al. performed microarray experiments investigating the effect of GATA3 on MDA-MB-231 breast cancer cells (data accessible from the National Center for Biotechnology Information GEO database (Green et al, 2010), accession GSE24249) in 2012. We used online software GEO2R of the GEO database (www.ncbi.nlm.nih.gov/geo/geo2r) to analyze differentially expressed genes of this microarray across experimental conditions. The top 20 genes with the greatest variation in differential expression were selected as our candidate genes. Of these 20 genes, we focused on adhesion molecules or proteases. We downloaded the data on the entire ENTPD family from this database, and utilized software (MultiExperiment Viewer) to set up a heatmap that reflected the changes in ENTPD family members under different treatment conditions. Simultaneously, we made use of the online database, Gene expression-based Outcome for Breast cancer Online (GOBO) (http://co.bmc.lu.se/gobo/gsa.pl) to explore the correlation between ENTPD3 and the clinical-pathological characteristics of breast cancer. Furthermore, the Kaplan-Meier plotter tool (http://kmplot.com/analysis/index.php?p=background) was applied to probe whether ENTPD3 is a biomarker of survival. Also, a Cistrome Dataset Browser (DB; http://cistrome.org/db/#/) was utilized to analyze if GATA3 could bind to the ENTPD3 promoter.

### Cell culture and transfection

MDA-MB-231, MDA-MB-436, MDA-MB-453, BT20, BT549, HCC1937, T47D, MCF10A, and MCF-7 cell lines were purchased from the American Type Culture Collection and cultured in standard conditions as recommended. Small interfering (si)RNAs targeting ENTPD3 or GATA3 as well as negative controls were synthesized by Suzhou GenePharma (Suzhou, China). The sequences of the siRNAs are displayed in **Table S1**. High-expressing ENTPD3 and negative control plasmids were obtained from Sino Biological (HG10909-NF; Wayne, PA, USA). The CDS of GATA3 was cloned into a pCDNA3.1 vector to establish a high-expressing GATA3 plasmid as previously described 11. We utilized Lipofectamine 3000 agent to transfect cells with siRNAs or plasmids according to the manufacturer's instructions.

### RNA extraction, reverse transcription, and real-time qPCR

We used an adsorption column for the separation of total cellular mRNA using a MiniBEST Universal RNA Extraction Kit (Takara, Beijing, China). A PrimeScript™ RT Master Mix (Perfect Real Time) Kit (Takara) was used to synthesize cDNA. Real-time quantitative (RT-q)PCR was performed with a SYBR Select Master Mix (Thermo Fisher Scientific, Waltham, MA, USA) using a CFX96 Real-time PCR Detection System machine (Bio-Rad, Hercules, CA, USA). ENTPD3 primers were purchased from Sino Biological (HP100931). The primer for the amplification of GATA3 was designed as follows: forward, AGCCACTCCTACATGGACGC; and reverse, AAGGGGCTGATTCCAGGG. We normalized Ct values to beta-actin and calculated relative expression using a -2^ΔΔCt^ method.

### Western blotting

Cells were lysed in RIPA buffer plus phenylmethylsulfonyl fluoride on ice for 10 min. Lysates were exposed to ultrasound waves three times (4 sec per exposure) and centrifuged at 12,000 rpm for 15 min to remove insoluble material. A BCA Protein Assay Kit (Thermo Scientific) was used to measure protein concentration. After denaturation, 40 μg of protein was subjected to SDS-polyacrylamide gel electrophoresis and transferred to polyvinylidene difluoride membranes, blocked in 5% milk, incubated overnight with primary antibody followed by the addition of secondary antibody. Antibodies used are listed in **Table S2**. ECL Detection Reagents (Thermo Scientific) were used to visualize the immunoblots.

### Chromatin immunoprecipitation assay

MCF-7 cells were grown to 90% confluence in 100-mm Petri dishes for the chromatin immunoprecipitation (ChIP) assay. The *ENTPD3* promoter sequence was immunoprecipitated using a ChIP Assay Kit obtained from Beyotime (Shanghai, China) following the manufacturer's protocol. A ChIP grade GATA3 antibody (Abcam, ab199428) was utilized in the experimental group. PCR was used to confirm results. The primers for PCR amplification were as follows: forward, 5'GGCCTCACTCCCAAC ATTAC3'; and reverse, 5'CTGCCTCCTTCTTGCATCTG3', generating a 212-bp PCR product containing the GATA3 binding site.

### Dual-luciferase reporter assay

The *ENTPD3* promoter sequence (from -1525 to -33 bp) was cloned into a pGL3-enhancer vector (Panomics, Fremont, CA, USA) between SacI and Smal sites. The primers for amplifying the targeted sequence were designed as follows: forward, CGAGCTCGGGTCCACCTCTATCCAA; and reverse, TCCCCCGGGG GGCTACCGTGTTTCAGT. To standardize transfection efficiency, a pRL-SV40 vector (Promega Madison, WI, USA; E2231) was used as the control vector and transfected into cells at the same time. We used a ONE_Glo EX Luciferase Assay System kit (Promega; E8110) to detect luciferase activity according to the manufacturer`s protocol.

### Immunohistochemistry

We randomly chose 27 triple negative and 19 luminal A subtype breast cancer patient samples from our tissue bank that were collected in 2014 or 2015 (**Table S3**). Immunohistochemistry (IHC) was performed using the standard protocol. The entire procedure included sectioning, heating sections, dewaxing, the restoration of antigens, blocking, incubation with primary antibody overnight, the quenching of endogenous peroxidase, incubation with secondary antibody, staining, and sealing. The primary antibodies used were the same as those used in western blotting. The secondary antibody was purchased from MXB Biotechnologies (Fuzhou, China). IHC scoring depended on the proportion of cells showing positive-staining intensities. We defined proportion scores as follows: 0 (no staining of cells), 1 (≤25%), 2 (>25% and ≤50%), or 3 (>50%). The intensity scoring was defined as 0 (no staining), 1 (light yellow), 2 (yellow), or 3 (brown). The final scores were expressed as intensity scores: low (≤3), intermediate (3> and ≤6) or high (>6). Every slice was examined by three independent pathologists.

### Transwell migration and colony formation assays

#### Migration/invasion assay

Chambers with 8-μM pore membranes (BD, Franklin Lakes, NJ, USA; 353097/354480) were used in Transwell migration/invasion assays. First, cells were cultured in medium without fetal bovine serum for 24 h. Cells were then digested with pancreatin and diluted in blank medium. The chambers were inoculated with cells (MCF-7, 2×10^4^ and MDA-MB-231, 1×10^4^). Complete medium was added to the bottom chamber. After 24 or 48 h, migrated cells were stained with 0.1% crystal violet. To test the pro-migration/invasion effect of eATP, ATP (Sigma-Aldrich, St Louis, MI, USA; 34369-07-8) was added to the upper or lower chamber of each Transwell. PBS served as a negative control.

#### Colony formation assay

After digestion, cells were diluted with complete medium. A cell counter was used to measure cell numbers. Cells (0.5×10^3^) were seeded in 6-well plates and cultured for 14 days. Cells were fixed with methanol and stained with 0.1% crystal violet. To verify whether eATP improved cell stemness, ATP was used as the experimental agent and PBS served as a negative control.

#### ATPase activity assay

An ATPase/GTPase Activity Assay Kit was obtained from Sigma-Aldrich (MAK113). ATPases catalyze ATP into ADP and release free phosphate. Malachite green reagent and free phosphate can form a stable dark green color that shows maximum light absorbance at 620 nm. The ATPase Activity Assay was performed according to the manufacturer's protocol. We utilized released inorganic phosphate (micromoles per minute per milligram) to reflect ATPase activity. Each assay was performed in triplicate.

### Measurement of ADP/ATP ratio and ATP degradation rate

An ADP/ATP Ratio Assay Kit (Sigma-Aldrich; MAK135) was used to measure the ADP/ATP ratio and ATP degradation rate. First, we set up a standard curve of relative light units and ATP concentration. The same number of cells was seeded in 6-well plates. After adhesion, cells were cultured in serum-free medium instead of total medium, and ATP was added to the medium to a final concentration of 200 μM. Medium samples (10 μL) were collected at 0, 1, and 2 h. The ATP concentration of the medium was tested at three different time points to set up an ATP hydrolytic rate curve. The ADP/ATP ratio was tested at 1 h. All experimental procedures were undertaken according to the manufacturer's technical bulletin. Each assay was performed in triplicate.

### Site-directed mutagenesis

A Fast Mutagenesis System Kit was purchased from Transgene Biotech (Beijing, China) and used to perform site-directed mutagenesis. The mutagenesis of wild-type *ENTPD3* in a pCMV3-N-flag vector was carried out according to the manufacturer's protocol. The primers for PCR amplification were as follows: forward, GGACTTAGGTGGTGCCGCCACCCAAATATCC; and reverse, CGGCACCACCTAAGTCCAGGGCAACCCGT. After the extraction of mutated plasmids, we confirmed sequences by next generation sequencing. Mutagenesis of the wild-type *ENTPD3* promoter in the pGL3-enhancer vector was similar to that for wild-type *ENTPD3*. The primers were designed as follows: forward, 5'GCAAGAAGGAGGCATATAATAGACACCTCCC3'; and reverse, 5'ATGCCTCCTTCTTGCATCTGTGTCAAGACCAAG3'.

### Animal experiments

All animal experiments were approved by the Institutional Animal Care and Use Committee of Shantou University Medical College (SUMC). Five- to six-week-old female NOD-SCID mice were purchased from Vital River Laboratories (Beijing, China). All mice were housed in pathogen-free conditions in the animal experiment center of SUMC. MDA-MB-231-control/ ENTPD3/M224 cells (2 × 10^6^) were randomly injected into each NOD-SCID mouse via a tail vein. Three weeks later, an IVIS Kinetic Imaging System (PerkinElmer, Waltham, MA, USA) was used to monitor distant organ metastases or primary tumors once a week. Six weeks after the injection, all mice were euthanized, and tumors and metastatic nodes in the lungs counted.

### Statistical analysis

We used SPSS Software (20.0) to analyze statistical differences. A two-tailed Student's *t*-test or one-way analysis of variance test was used. Spearman's rank correlation coefficient test was performed to verify correlation. Fisher exact test was carried out to compare the lung metastatic ratio. We considered *p* < 0.05 as significant.

## Results

### GATA3 up-regulated expression of ENTPD3

**Figure 1:**
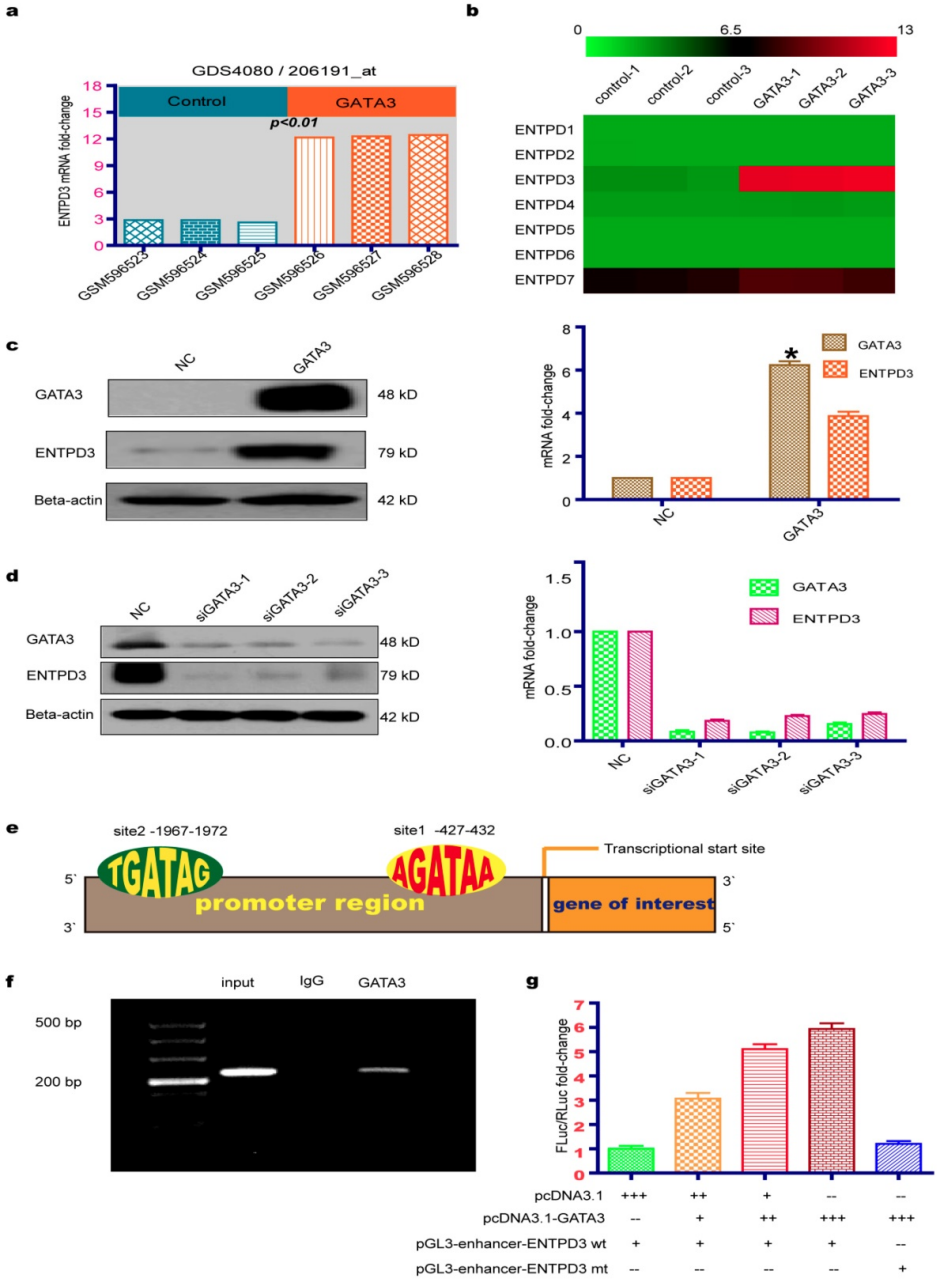
** GATA3 up-regulates ENTPD3 expression.** (a) Relative *ENTPD3* mRNA levels from a gene expression omnibus (GEO) microarray in MDA-MB-231 breast cancer cell lines that were transfected with lentivirus expressing GATA binding protein 3 (GATA3). (b) A thermal map from GEO microarrays of the effect of GATA3 ectopic expression on the ectonucleoside triphosphate diphosphohydrolase (*ENTPD*) family in MDA-MB-231 breast cancer cells. (c) Relative *ENTPD3* mRNA (real-time [RT]-quantitative [q]PCR) and protein (western blot) levels in MDA-MB-231 breast cancer cells that were transfected with *GATA3*-expressing plasmids (* N×100). (d) Relative* ENTPD3* mRNA (RT-qPCR) and protein (western blot) levels in MCF-7 cells in which GATA3 was knocked down by small interfering (si)RNAs. (e) The GATA3-binding site location of the *ENTPD3* gene. (f) Chromatin immunoprecipitation (ChIP)-PCR assay. (g) Dual-luciferase reporter assay (-- 0 pmole, + 10 pmole, ++ 20 pmole, +++ 40 pmole, wt wild type, mt mutant type). NC, negative control. Error bars, s.e.m. Repeated three times for each experiment.

Microarray results from a GEO database (GSE24249) provided insight into the molecular basis of the GATA3-mediated reduction of TNBC metastasis. We utilized the GEO GEO2R tool to analyze differentially expressed genes between MDA-MB-231 breast cancer cell lines that overexpressed or did not overexpress GATA3. By analyzing the functions of 20 genes with the greatest variation in differential expression, we discovered that ENTPD3 may be a key downstream molecular regulator of GATA3 that inhibits breast cancer metastases. Interestingly, we found that the overexpression of *GATA3* in MDA-MB-231 cells led to significantly increased *ENTPD3* mRNA expression (adjusted *p*-value < 0.01; **Fig. 1a**). After downloading the corrected raw data to set up the heatmap, we found that the mRNA of *ENTPD* family was not expressed in MDA-MB-231 cells, except for *ENTPD7*, and that GATA3 merely increased the expression of *ENTPD3* (**Fig. 1b**). Our data are consistent with GEO microarray data at mRNA and protein levels (**Fig. 1c**). To analyze the effect of GATA3 on *ENTPD3* expression, we used siRNAs to silence *GATA3* expression in MCF-7 cells. Interestingly, the inhibition of GATA3 lowered *ENTPD3* expression (**Fig. 1d**). By searching the promoter sequence, we found two GATA3-binding sites in the promoter of *ENTPD3*, which were located between -427 and -432, close to the transcriptional start site, and between -1967 and -1972 (**Fig. 1e**). Moreover, in the Cistrome DB ChIP-sequencing (Seq) database, we found that a GATA3 antibody pulled down the *ENTPD3* promoter sequence in MCF-7 cells. In this study, when we performed a ChIP-PCR experiment, the GATA3 antibody, but not the control IgG, specifically pulled down the *ENTPD3* promoter in MCF-7 cells (**Fig. 1f**). The primers amplifying the promoter were designed to cover the binding site adjacent to the transcriptional start site. Dual-luciferase reporter assays demonstrated that GATA3 boosted the activity of the *ENTPD3*-promoter reporter and that deleting the GATA3-binding site diminished GATA3-mediated activity in MDA-MB-231 breast cancer cells (**Fig. 1g**).

### GATA3 accelerated eATP hydrolysis mediated by ENTPD3 and partially reversed the pro-metastatic effect of eATP

**Figure 2:**
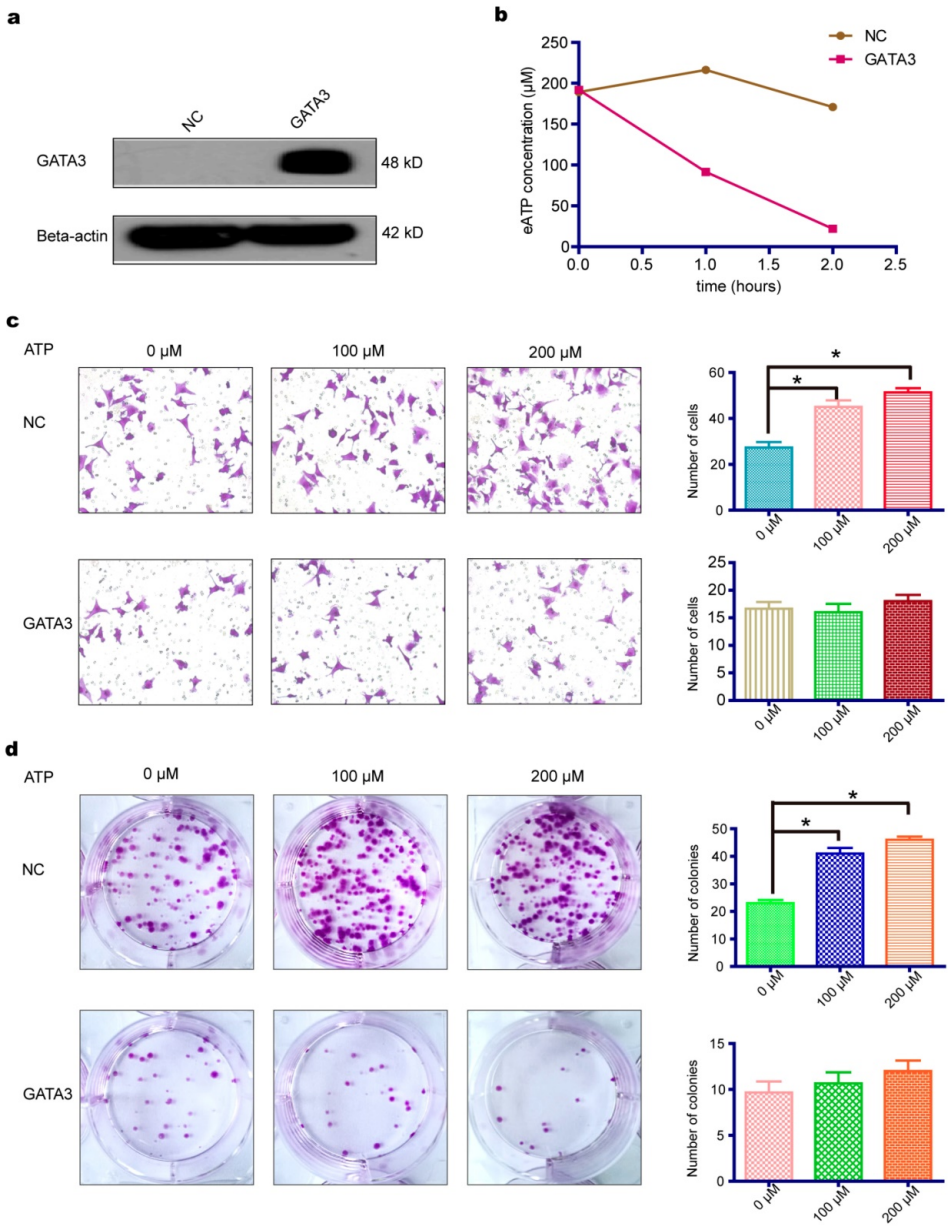
** GATA3 promotes hydrolysis of eATP and partially reverses its pro-metastatic characteristic.** (a) Effect of GATA binding protein 3 (GATA3) plasmid transfection in MDA-MB-231 breast cancer cells as shown by western blotting. (b) Hydrolysis rate of extracellular (e)ATP. (c) MDA-MB-231 cell migration assay with different doses of eATP (*p < 0.05, *t*-test). (d) Colony formation assay of MDA-MB-231 cells with different doses of eATP (*p < 0.05, *t*-test). NC, negative control. Error bars, s.e.m. Repeated three times for each experiment.

**Figure 3:**
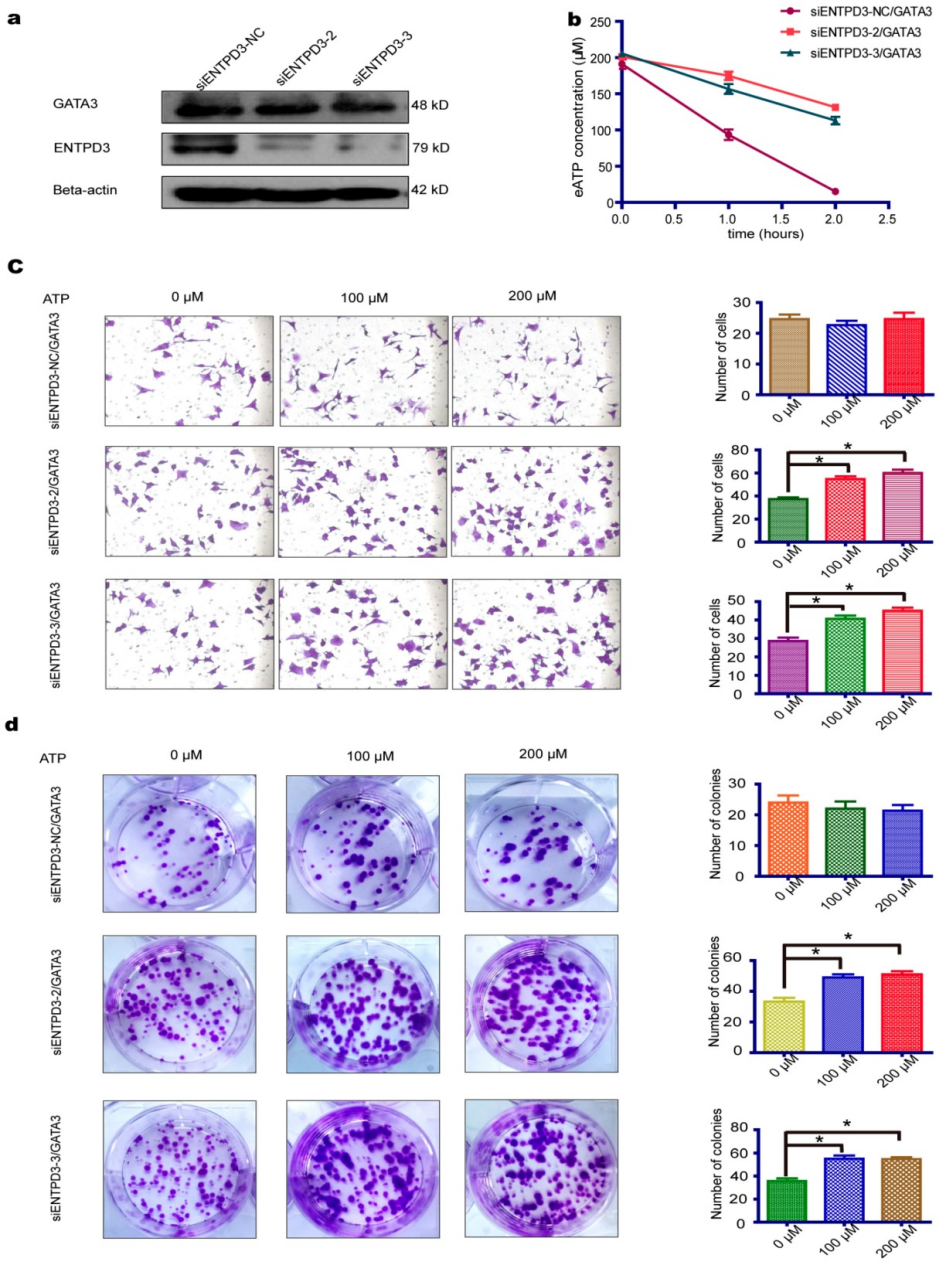
** ENTPD3 is indispensable for mediating eATP hydrolysis by GATA3.** (a) Western blots of lysates from GATA binding protein 3 (GATA3)-overexpressing and ectonucleoside triphosphate diphosphohydrolase (ENTPD3) knockdown MDA-MB-231 cells. (b) Extracellular (e)ATP hydrolysis curve. (c) Migration of MDA-MB-231 cells treated with different doses of eATP (*p < 0.05, *t*-test). (d) Colony formation by MDA-MB-231 cells treated with different doses of eATP (*p < 0.05, *t*-test). NC, negative control. siRNA, small interfering RNA. Error bars, s.e.m. Repeated three times for each experiment.

As shown above, GATA3 enhanced the expression of ENTPD3, an ectonucleotidase that hydrolyzes eATP. We, therefore, tested whether the overexpression of GATA3 accelerated the hydrolysis of eATP in MDA-MB-231 cells (**Fig. 2a**). As expected, the overexpression of GATA3 resulted in increased eATP hydrolysis (**Fig. 2b**). Next, we evaluated if GATA3 affected eATP functions. By adding different concentrations of ATP to the cell culture medium, we discovered that eATP promoted migration and colony formation by MDA-MB-231 cells and that the eATP-induced progression of breast cancer was partially reversed by the increased expression of GATA3 (**Fig. 2c,d**). The above results demonstrated that GATA3 up-regulated *ENTPD3* and boosted the hydrolysis of eATP. However, a direct association between GATA3-accelerated eATP hydrolysis and ENTPD3 was not clear. To show a direct link, we decreased ENTPD3, using various siRNAs, in MDA-MB-231 cells that stably expressed GATA3. Only two siRNAs targeting* ENTPD3* reduced its expression (**Fig. S1**). After successfully reducing the expression of ENTPD3 (**Fig. 3a**), we tested the hydrolysis rate of eATP. Compared with the negative control, decreasing ENTPD3 led to a decline in the eATP hydrolysis rate (**Fig. 3b**). Furthermore, decreased ENTPD3 levels partially reversed the GATA3-mediated inhibition of eATP-induced migration (**Fig. 3c**). A similar phenomenon was observed in the colony formation assay. When we silenced the expression of ENTPD3, the GATA3-mediated inhibition of eATP-induced colony formation was reduced (**Fig. 3d**). In addition, as shown in **Fig. S2**, the vimentin protein level declined and E-cadherin protein level increased when MDA-MB-231 cells stably expressed GATA3; meanwhile, when we reduced ENTPD3 level by siRNA, the expression of E-cadherin remained unchanged, while expression of vimentin increased slightly.

### ENTPD3 inhibited breast cancer progression by hydrolyzing eATP *in vitro*

**Figure 4:**
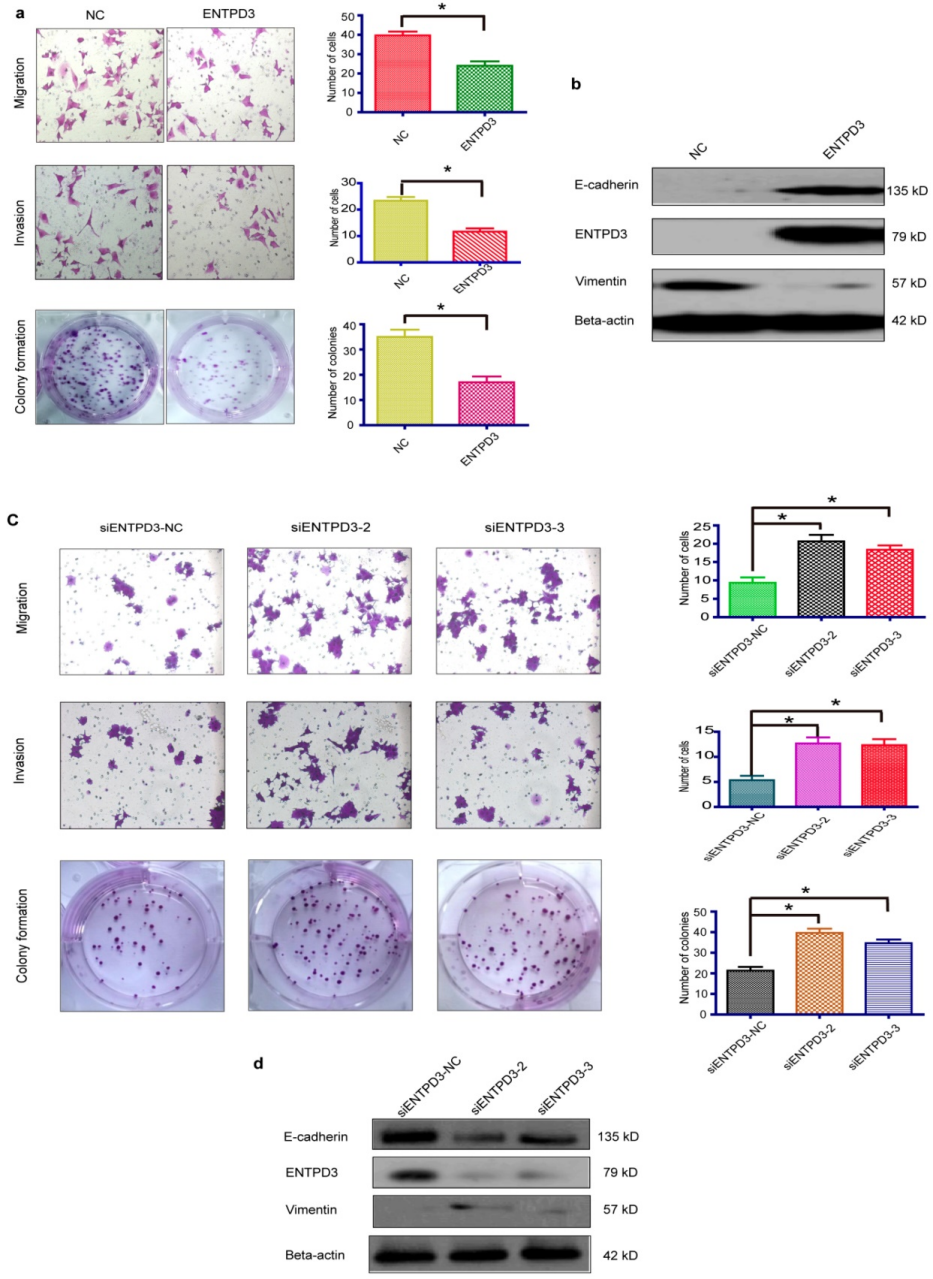
** ENTPD3 inhibits breast cancer progression.** (a) Migration/invasion and colony formation assays in MDA-MB-231 cells transfected with ectonucleoside triphosphate diphosphohydrolase 3 (ENTPD3) plasmids (*p < 0.05,* t*-test). (b) Alteration in vimentin and E-cadherin levels in MDA-MB-231 cells transfected with *ENTPD3* plasmids (western blots). (c) Migration/invasion and colony formation assays in MCF-7 cells in which ENTPD3 was knocked down by small interfering (si)RNAs (*p < 0.05, *t*-test). (d) Modification of vimentin and E-cadherin protein levels as determined by western blotting in MCF-7 cells in which ENTPD3 was knocked down by siRNAs. NC, negative control. Error bars, s.e.m. Repeated three times for each experiment.

**Figure 5:**
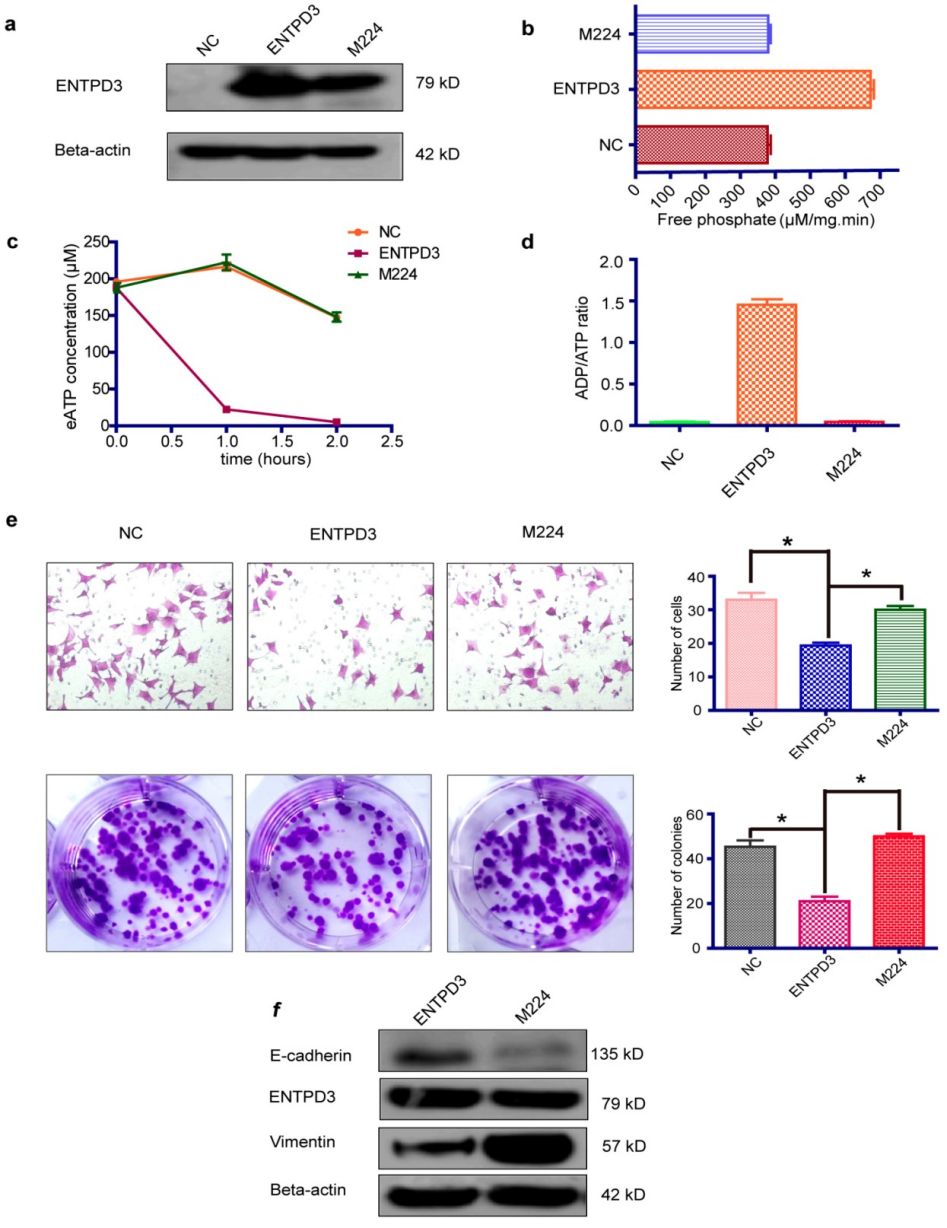
** ENTPD3 inhibits breast cancer metastasis by hydrolyzing eATP.** (a) Relative ectonucleoside triphosphate diphosphohydrolase 3 (ENTPD3) protein levels as determined by western blots in MDA-MB-231 cells transfected with ENTPD3 or M224 plasmids (M224 mutagenesis of ENTPD3 serine 224 to alanine). (b) ATPase activity (μM/mg.min; releasing μmoles of free phosphate per mg protein per minute under assay conditions). (c) Extracellular (e) ATP hydrolysis curve. (d) ADP/ATP ratio analysis when ENTPD3 converts ATP to AMP. (e) Migration and colony formation assays of MDA-MB-231 cells (*p < 0.05, *t*-test). (f) Alteration of vimentin and E-cadherin levels in MDA-MB-231 cells transfected with the M224 plasmid (western blots). NC, negative control. Error bars, s.e.m. Repeated three times for each experiment.

It is widely accepted that luminal subtype breast cancer cells are lower migration capability than TNBC. Interestingly, we discovered that ENTPD3 was expressed at an extremely higher level in MCF-7, compared to MDA-MB-231 cells (**Fig. S3a**). ENTPD3 is an ectonucleotidase. We, therefore, tested the difference in ATPase activity between MCF-7 and MDA-MB-231 cells. As expected, MCF-7 cells hydrolyzed eATP faster than MDA-MB-231 cells (**Fig. S3b**). Additionally, the overexpression of ENTPD3 not only reduced cell migration/invasion but also reduced cell colony formation by MDA-MB-231 cells (**Fig. 4a**). Furthermore, the vimentin protein level declined and E-cadherin protein level increased when MDA-MB-231 cells were transfected with an *ENTPD3* plasmid (**Fig. 4b**). Conversely, when we silenced *ENTPD3* expression in MCF-7 cells using siRNAs, cellular migration/invasion and colony formation were promoted (**Fig. 4c**), while E-cadherin and vimentin expression mildly decreased and increased, respectively (**Fig. 4d**). A previous study demonstrated how the mutagenesis of ENTPD3 serine (224) to alanine (M224) resulted in decreasing the expression and loss of hydrolase activity in COS-1 cells 29. To test whether the inhibition of tumors is mediated by the activity of the ENTPD3's ATPase, we used the same method to eliminate or weaken the enzymatic activity of ENTPD3 in MDA-MB-231 breast cancer cells. Interestingly, M224 also led to the decreased expression (**Fig. 5a**) and loss of hydrolase activity (**Fig. 5b**) in MDA-MB-231 cells. Meanwhile, we tested the eATP hydrolysis rate to determine whether M224 led to a loss of hydrolase activity by ENTPD3. As expected, the eATP degradation curve of the M224 group was similar to that of the negative control group in MDA-MB-231 cells (**Fig. 5c**). When ENTPD3 converted ATP to AMP, the accumulation of ADP was observed (**Fig. 5d**). Furthermore, as compared to the negative control, the overexpression of wild-type ENTPD3 led to decreased migration and colony formation by MDA-MB-231 cells, but not the mutation group (**Fig. 5e**). In addition, the mutation group did not show the up-regulated expression of E-cadherin and down-regulated expression of vimentin (**Fig. 5f**). Moreover, wild-type ENTPD3 eliminated the pro-metastatic ability of eATP in MDA-MB-231 cells, while the mutation group was devoid of this property (**Fig. S4a, b**).

### ENTPD3 inhibited breast cancer progression in an immunodeficient mouse model

**Figure 6:**
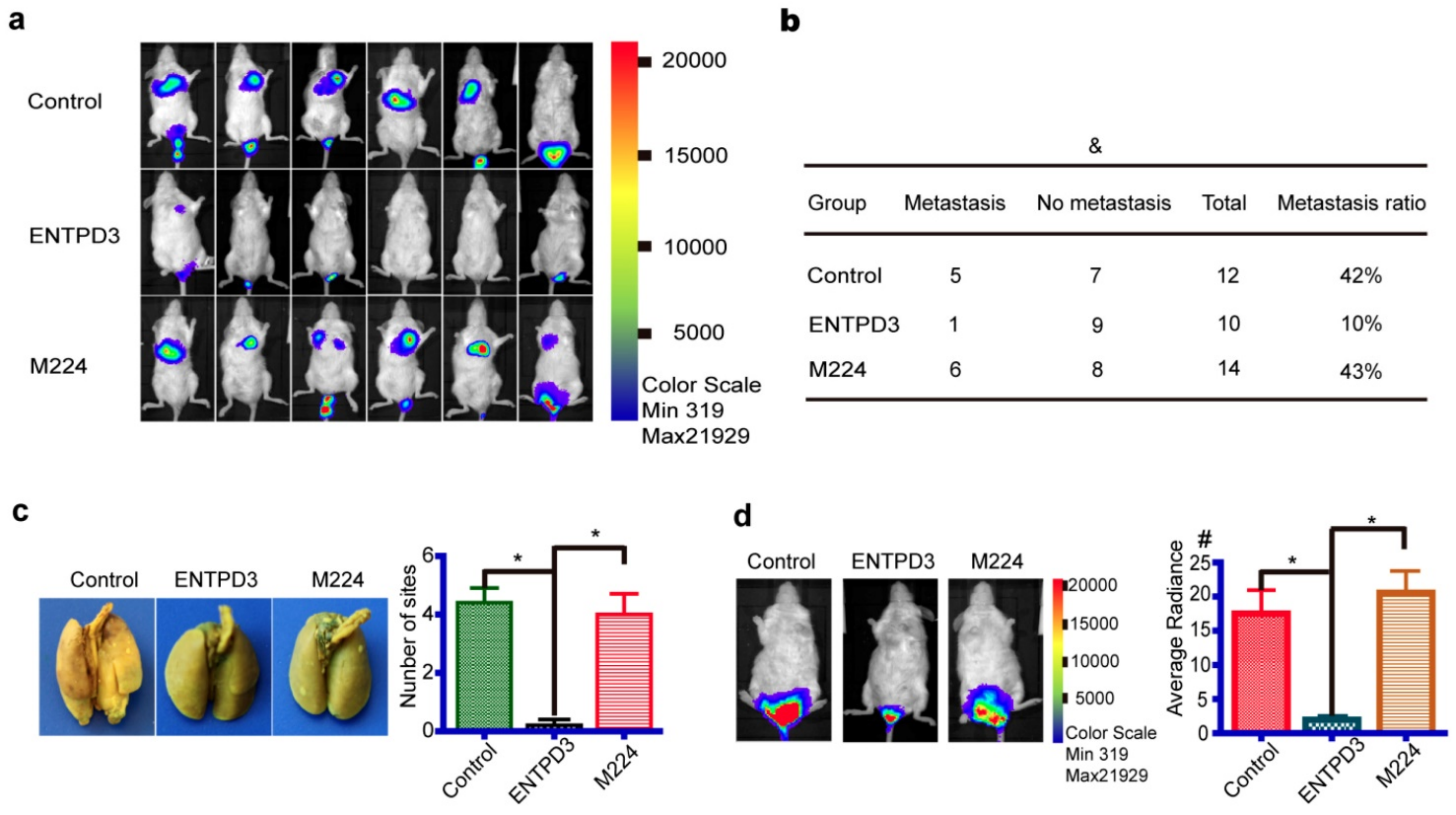
** ENTPD3 inhibits breast cancer cell metastasis in NOD-SCID mice.** (a) Representative bioluminescence images of lung metastases six weeks after injection (sample size: Control 12, ENTPD3 10, M224 14). (b) The metastasis ratio in the lung in different groups six weeks after injection (^&^*p* > 0.05, Fisher exact test). (c) Representative pulmonary perfusions revealed different number of lung metastatic sites (*p < 0.05, *t*-test). (d) Average radiance six weeks after injection (unit, p/sec/cm2/sr; ^#^×10^7^; *p < 0.05, *t*-test). Error bars, s.e.m.

To investigate the antitumor role of ENTPD3 *in vivo*, we injected MDA-MB-231 cells stably expressing either wild-type ENTPD3 or mutant M224, or control plasmids into NOD-SCID mice via tail veins. The cells were also labeled with luciferase so that they could be used for *in vivo* imaging using an IVIS Kinetic Imaging System. Significantly, tumors grew in the lungs or buttocks of all control or mutation M224 group mice; however, three mice in ENTPD3 groups showed no tumor formation (**Fig. S5**). We detected bioluminescent signals in the lungs of six mice from the M224 group, five mice from the control group, but only one mouse from the ENTPD3 group (**Fig. 6a**). The lung metastasis rate was 10% in the ENTPD3 group, 42% in the control group and 43% in the M224 group (**Fig. 6b**). Nevertheless, a statistical significance between these groups was not found and we speculated that limited sample numbers was the cause. In addition, pulmonary metastatic sites were more numerous in control/M224 groups than in the ENTPD3 group (**Fig. 6c**). Furthermore, wild-type ENTPD3 suppressed tumor growth in the buttocks of mice that did not grow tumors in their lungs (**Fig. 6d**).

### ENTPD3 was a favorable prognostic factor in breast cancer patients

**Figure 7:**
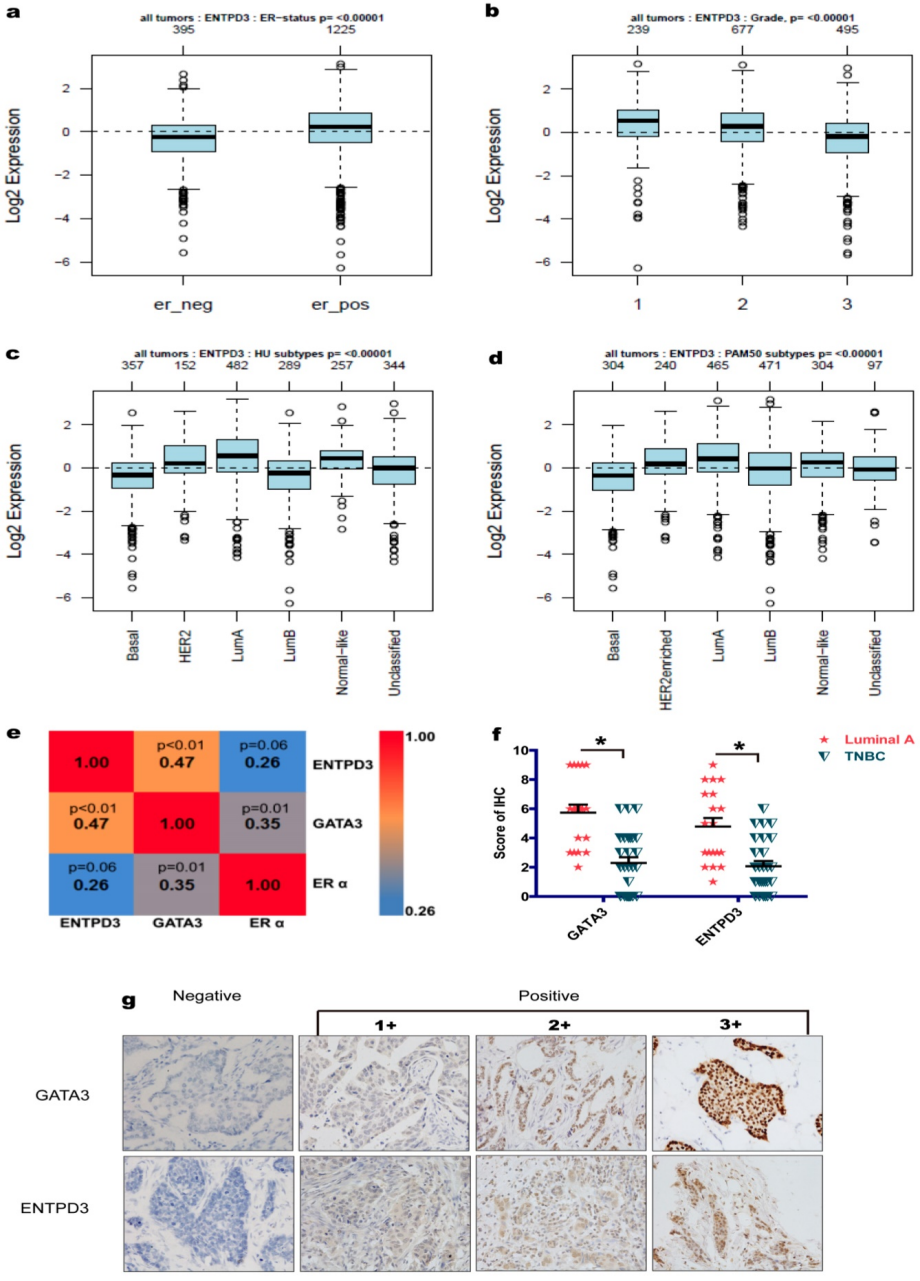
** ENTPD3 is enriched in the luminal subtype.** (a) Correlation between estrogen receptor α (ERα) and ectonucleoside triphosphate diphosphohydrolase 3 (ENTPD3) mRNA levels from the Gene expression-based Outcome for Breast cancer Online (GOBO) database. (b) Correlation between ENTPD3 mRNA levels and histopathological tumor grade from the GOBO database. (c,d) ENTPD3 mRNA levels in different molecular subtypes of breast cancer in the GOBO database. Central value, median. (e) Coefficient matrix heat map of ENTPD3, GATA binding protein 3 (GATA3) and ERα from immunohistochemistry (IHC) results (Spearman rank tests). (f) GATA3 or ENTPD3 protein level distribution in luminal A and triple negative breast cancer (TNBC) from IHC results (*p < 0.05, nonparametric tests). (g) Representative IHC images of ENTPD3 and GATA3 stains in patients with breast cancer. Error bars, s.e.m.

In the GOBO database, the *ENTPD3* mRNA level was positively correlated to estrogen receptor α (ERα) and negatively correlated to tumor histopathological grade in patients with breast cancer (**Fig. 7a, b**). IHC revealed that ENTPD3 was localized to breast cancer cell membranes and the cytoplasm, and showed a positive correlation with ERα (**Fig. 7e, g**). Also, we showed that GATA3 and ENTPD3 were co-expressed in breast cancer at the protein level (**Fig. 7e**). In addition, ENTPD3 expression in the luminal A subtype was highest among all breast cancer molecular subtypes (**Fig. 7c, d**). IHC also showed that luminal A patients expressed higher GATA3 and ENTPD3 level than patients with TNBC (**Fig. 7f**). The Kaplan-Meier plotter tool was used to assess the effect of 54,675 genes on survival using 5,134 breast cancer samples. In this database, data from 3,951 cases was available for the analysis of the correlation between *ENTPD3* expression and recurrence-free survival (RFS). In terms of overall survival (OS), 1,402 patients provided usable information. The cut-off value was designed as the upper tertiles. We found that ENTPD3 was a favorable prognostic factor in patients with breast cancer, either RFS or OS (**Fig. S6a, b**).

## Discussion

**Figure 8:**
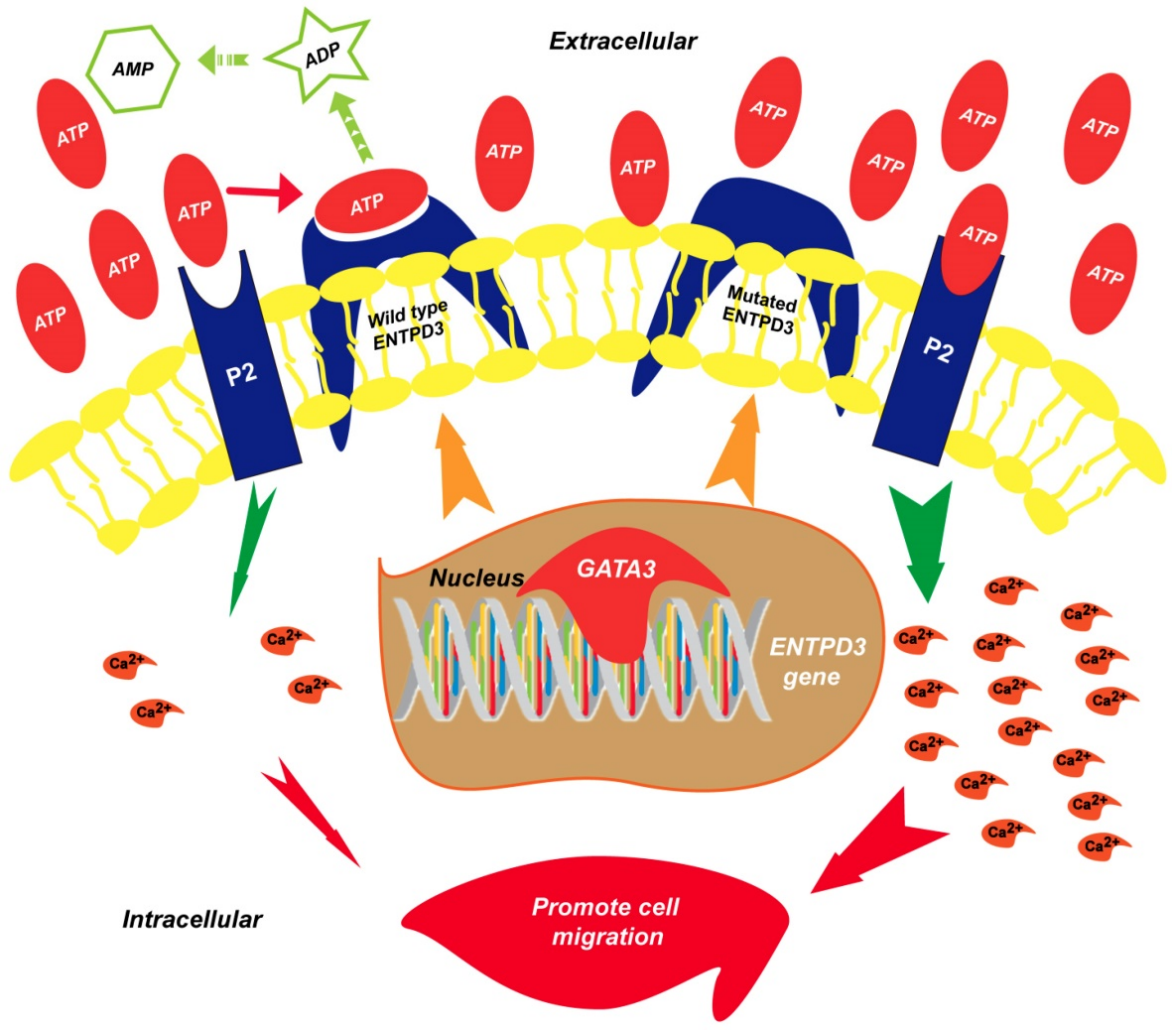
** Proposed model of the suppression of breast cancer metastasis via GATA3 by up-regulating ENTPD3.** GATA binding protein 3 (GATA3) up-regulates ectonucleoside triphosphate diphosphohydrolase 3 (ENTPD3) by binding to the promoter of *ENTPD3*. If the *ENTPD3* gene is wild type, GATA3-mediated ENTPD3 reduces eATP-induced Ca^2+^ influx and suppresses breast cancer metastasis by hydrolyzing eATP. Otherwise, GATA3 could not inhibit the metastasis of breast cancer by up-regulating the ENTPD3 pathway due to the loss of ATPase activity in ENTPD3 caused by a mutation.

In this study, we demonstrated for the first time that ENTPD3 was a novel GATA3-regulated protein. Specifically, GATA3 accelerated the hydrolysis of eATP and suppressed breast cancer cell metastasis by up-regulating ENTPD3 (**Fig. 8**). Furthermore, GATA3 up-regulated ENTPD3 expression by binding to the *ENTPD3* promoter. The ENTPD3-mediated hydrolysis of eATP in the cancer microenvironment suppressed breast cancer cell dissemination and served as a tumor suppressor. Thus, our findings provide novel insights into the GATA3's inhibition of breast cancer metastasis.

GATA3 is a key transcription factor that is indispensable in organ development, such as for mammary glands, kidneys, and skin 30-33. In transgenic mice models, the loss of GATA3 led to the failure of mammary-gland morphogenesis 5-7, 30. Utilizing a doxycycline-inducible system, GATA3 was conditionally deleted in the adult mouse mammary gland following which luminal epithelial cells showed de-differentiation, decreased cell-cell adhesion and increased cellular proliferation 30. These observations highlight the significance of GATA3 during breast development and the maintenance of function. Cancer Genome Atlas data showed that about 10% of patients with breast cancer harbored mutations in the *GATA3* gene 34, 35. As previously reported, GATA3 was expressed in ERα-positive patients at an early stage but was lost by the time their cancers became advanced 36. Furthermore, the expression of GATA3 was always absent in patients with TNBC 8. Yan et al. confirmed that GATA3 decreased cancer cell motility by inhibiting EMT 9. In particular, cancer metastasis is a complicated process involving a significant number of molecular events. GATA3 acts as a pleiotropic modulator of the molecules that are indirect or are immediate effectors of various cellular processes that repress breast cancer metastasis. For example, Chou et al*.* reported that GATA3 modulated the breast cancer microenvironment and inhibited metastasis through the up-regulation of microRNA-29b, which repressed the expression of multiple genes, including *VEGFA*, *ANGPTL4*, *PDGF*, *LOX*, and *MMP9* 10. The present study showed that GATA3 blocked breast cancer cell motility by up-regulating ENTPD3 expression, which in turn hydrolyzed eATP in the tumor microenvironment. This provides additional evidence supporting the viewpoint that GATA3 restrains breast cancer metastasis by altering the tumor microenvironment. Moreover, previous studies demonstrated that eATP was able to induce immune suppression and prevent the recognition of malignant cells as foreign entities 14, 21, 37. GATA3 improves the anti-cancer ability of T lymphocytes in the tumor microenvironment by the ENTPD3-catalyzed hydrolysis of eATP. Thus, the up-regulation of ENTPD3-mediated GATA3 not only inhibits breast cancer cell motility, but also modulates the tumor microenvironment to repress breast cancer cell metastasis. Our study has identified a novel effector of GATA3, ENTPD3, and provides a distinct perspective on the mechanism involved the GATA3 suppression of breast cancer progression and metastases.

So far, eight ENTPD family members have been identified in mammals 38. At a physiological extracellular pH between 7 and 8, such enzymes convert ATP and ADP into AMP in the presence of Ca^2+^ or Mg^2+^ 39. ENTPD1-3 and ENTPD8 are enzymes typically localized to the cell surface 40, while ENTPD4-7 are located in the membranes of intracellular organelles. Kinetic properties differ for ENTPD1-3 and ENTPD8. ENTPD2-3 and ENTPD8, especially ENTPD2, have a higher affinity for ATP than ADP 41. This feature leads to the accumulation of ADP when these enzymes hydrolyze ATP. However, ATP is hydrolyzed by ENTPD1 directly to AMP without the formation of an intermediate product, ADP 42. Our results further confirmed the hydrolysis of ATP to ADP by ENTPD3. Previous studies have described the aberrant function of ENTPD3 in human Crohn's, Parkinson's, and Alzheimer's diseases 43-45. However, the role of ENTPD3 has not yet been well defined in human cancers. In mouse models, the expression of ENTPD3 progressively decreased in bladder cancer, suggesting a potential role as a tumor suppressor 28. In human bladder cancer cell lines, the expression of ENTPD3 was present in RT4 cells having a low histological grade, but was absent in T24 cells with a high histological grade 46. This suggests that the absence of ENTPD3 expression is a marker of the malignant transformation in human bladder cancer cells. In the present study, we also found similar phenomena in human breast cancer cell lines. ENTPD3 was expressed in MCF-7 breast cancer cells with lower motility, but absent in MDA-MB-231 cells. Furthermore, we confirmed the above findings in patients with breast cancer: the expression of ENTPD3 in patients with luminal A cancer was higher than that in those with TNBC showing worse prognosis. Based on the above information, we conclude that *ENTPD3* is a suppressor gene for breast cancer. To further confirm the above viewpoint, we validated the role of ENTPD3 in breast cancer at the cellular and whole animal levels. We found that ENTPD3 inhibited the metastasis of breast cancer. Finally, we confirmed that the expression of ENTPD3 was a favorable biomarker of prognosis in a large sample of patients with breast cancer. Therefore, this study clarifies that ENTPD3 inhibits the metastasis and progression of breast cancer. ENTPD1, the most investigated member of the ENTPD family, repressed tumorigenesis and the progression of liver cancer via the degradation of eATP which boosted the Ras-mitogen-activated protein kinase pathway 47. In this study, by reducing the hydrolase activity of ENTPD3 through site-directed mutagenesis and observing the effect of the mutated ENTPD3 on cell processes at cellular and animal levels, we proved that ENTPD3 also inhibited the progression of breast cancer by degrading eATP. The decrease in E-cadherin and up-regulation of vimentin are the main markers of tumor cell EMT events. We demonstrated that ENTPD3 suppressed the expression of vimentin and promoted the expression of E-cadherin, suggesting that ENTPD3 inhibited breast cancer EMT. Previous studies revealed that EMT was calcium signaling-dependent and eATP stimulated calcium ion influx 18, 23 Thus, the finding on the hydrolysis of eATP by ENTPD3 indicates that ENTPD3 restrains EMT by hydrolyzing eATP in human breast cancer. Taken together, our findings support the notion that ENTPD3 is a tumor suppressor in breast cancers.

Extracellular ATP is a ubiquitous molecule in the extracellular matrix and is involved in diverse cellular responses by binding to P2 receptors 48. It was widely accepted that eATP was significantly higher in tumor tissue than in normal tissue by about 1000 times 49. However, discrepant results were reported about the significance of eATP in cancer cells 21. Extracellular ATP was first thought to activate the P2X7 receptor and act as a killer of cancer cells 50. However, increasing evidence confirmed that the killing of cancer cells by eATP was due to its overstimulation 21. As there was no technique available to accurately measure eATP concentration in normal or malignant tissues in the past, some researchers even used several hundred mmol/L-grade dose ATP to culture cancer cells. According to recent studies, P2X7R stimulation with an appropriate dose of ATP relevant to the tumor environment contributed to cancer cell invasiveness and metastatic distribution 18, 21, 51-55. Extracellular ATP binding to the P2X7 receptor was shown to trigger the activation of PI3K/Akt and ERK pathways that lead to cancer progression 53, 54. Also, a recent study pointed out that eATP could promote pancreatic cancer cell glycolysis by binding to P2Y2 56. Extracellular ATP induced breast cancer and fibroblast cells to release S100A4, which promoted cancer cell metastasis 57. Our study confirms that eATP promotes breast cancer metastasis. In addition, eATP in lung cancer tissues could be internalized by tumor cells, which not only promoted the growth and metastasis of cancer cells, but also induced drug resistance in cancer cells 58, 59. Taken together, we conclude that eATP improves the viability of cancer cells, and promotes the growth and metastasis of cancer cells in many ways, such as through energy metabolism, cytokines and cell signaling pathways. Furthermore, as we mentioned earlier, eATP also inhibits the function of lymphocytes in the tumor microenvironment, leading to the immunosuppression of the tumor microenvironment and promoting the escape of tumor cells from supervision and clearance by the body's immune system. Therefore, eATP provides a safe environment for the growth of cancer cells, which not only protects them from killing and clearing immune cells, but also provides energy, cytokines and other necessary material for their growth. Therefore, the specific reduction of eATP concentration in the tumor microenvironment is another new strategy for anti-cancer therapy, which is universal and suitable for all solid tumors and has extensive clinical applications.

In the present study, we demonstrated that ENTPD3 is a novel downstream effector of GATA3 and acts as a tumor suppressor in human breast cancer by hydrolyzing eATP. Chemotherapy and radiotherapy usually elevates the concentration of eATP in the tumor microenvironment 60, which may worsen therapeutic effects. An enhanced ENTPD3 level can hydrolyze eATP and inhibit cancer cell metastasis in those patients with lower ENTPD3 expression. In the future, a combination of traditional chemotherapy with strategies to augment ENTPD3 could be a potential strategy to improve breast cancer treatment.

## Supplementary Material

Supplementary figures and tables.

## Abbreviations

TNBC: triple negative breast cancer
EMT: epithelial-to-mesenchymal transition
eATP: extracellular ATP
ENTPD3: Ectonucleoside Triphosphate Diphosphohydrolase 3
ChIP: Chromatin immunoprecipitation
IHC: Immunohistochemistry
SUMC: Shantou University Medical College
M224: Mutagenesis of *ENTPD3* serine 224 to alanine
ERα: estrogen receptor α
RFS: recurrence-free survival
OS: overall survival
