# Supplementary material for: GATA Binding Protein 3 Boosts Extracellular ATP Hydrolysis and Inhibits Metastasis of Breast Cancer by Up-regulating Ectonucleoside Triphosphate Diphosphohydrolase 3
Source: Int J Biol Sci. 2019 Sep 7;15(12):2522–37. doi: 10.7150/ijbs.35563 (PMC6854379; doi:10.7150/ijbs.35563)
Supplement: Supplementary file 1 — Supplementary figures and tables. [file ijbsv15p2522s1.pdf]

# Supplementary

## Supplementary table

**Table.S1: Primer sequences of siRNAs**

| siRNAs | sense (5`-3`) | anti-sense (5`-3`) |
|---|---|---|
| siGATA3-1 | UCUGGAGGAGGAAUGCCAATT | UUGGCAUUCCUCCUCCAGATT |
| siGATA3-2 | ACGAGAAAGAGUGCCUCAATT | UUGAGGCACUCUUUCUCGUTT |
| siGATA3-3 | UCUGCUUCAUGGAUCCCUATT | UAGGGAUCCAUGAAGCAGATT |
| siENTPD3-1 | GCAUCCAAAGCUACUUCAATT | UUGAAGUAGCUUUGGAUGCTT |
| siENTPD3-2 | GCCAACUAUUUAAUGGGAATT | UUCCCAUUAAAUAGUUGGCTT |
| siENTPD3-3 | CCUCCACCCAAAUAUCCUUTT | AAGGAUAUUUGGGUGGAGGTT |
| Negative control | UUCUCCGAACGUGUCACGUTT | ACGUGACACGUUCGGAGAATT |

**Table. S2: Antibodies**

| Antibody | Manufacturer | Catalogue | Concentration |
|---|---|---|---|
| Beta actin | Abcam | ab6276 | 1/4000 |
| ENTPD3 | Invitrogen | PA5-24209 | 1/1000 |
| GATA3 | Abcam | ab199428 | 1/1000 |
| E-cadherin | Abcam | ab1416 | 1/1000 |
| Vimentin | Abcam | ab92547 | 1/1000 |
| Anti-mouse | Abcam | ab6728 | 1/1000 |
| Anti-rabbit | Abcam | ab6721 | 1/1000 |

**Table. S3: Patient's information**

| | Luminal A | Triple negative |
|---|---|---|
| Count | 19 | 27 |
| Median age | 49 | 47 |
| T1-2 | 15 | 15 |
| T3 | 4 | 12 |
| T4 | 0 | 0 |
| N0-1 | 16 | 20 |
| N2 | 3 | 7 |
| N3 | 0 | 0 |
| M0 | 19 | 27 |
| M1 | 0 | 0 |

## Supplementary figures

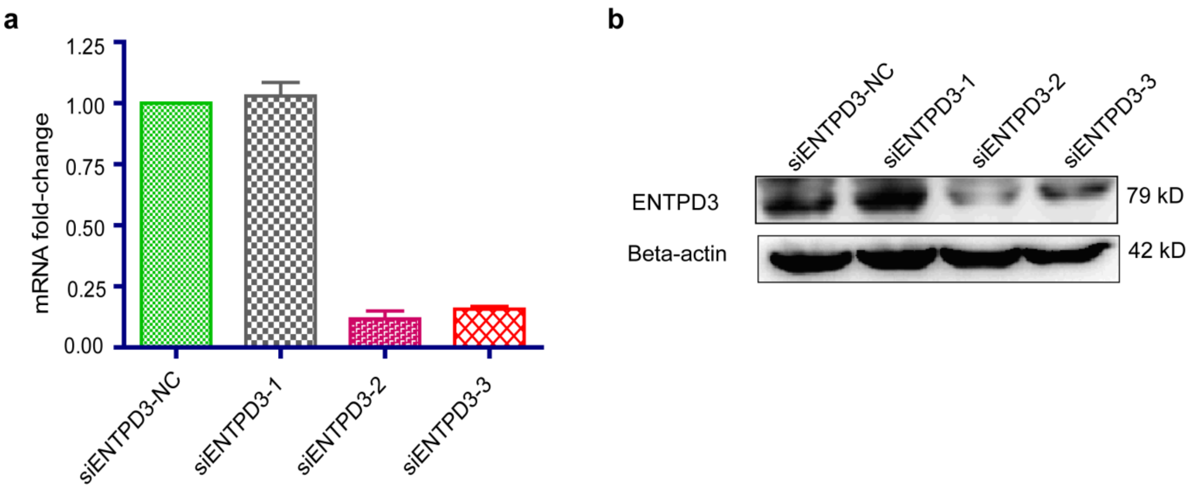


**Figure. S1: Interference effect of siRNAs targeting ENTPD3 in MCF-7 cells.** (a) Relative ENTPD3 mRNA (real-time–quantitative) level in MCF-7 cells in which ENTPD3 was knocked down by small interfering (si)RNAs. (b) Relative ENTPD3 protein (west-blotting) level in MCF-7 cells in which ENTPD3 was knocked down by small interfering (si)RNAs. Error bars, s.e.m. Repeated three times for each experiment.

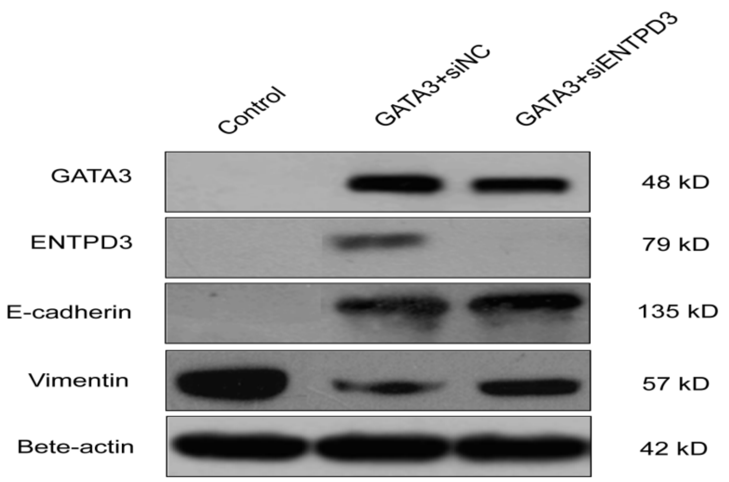

**Figure. S2: ENTPD3 rescue the effect of GATA3 on EMT makers.**

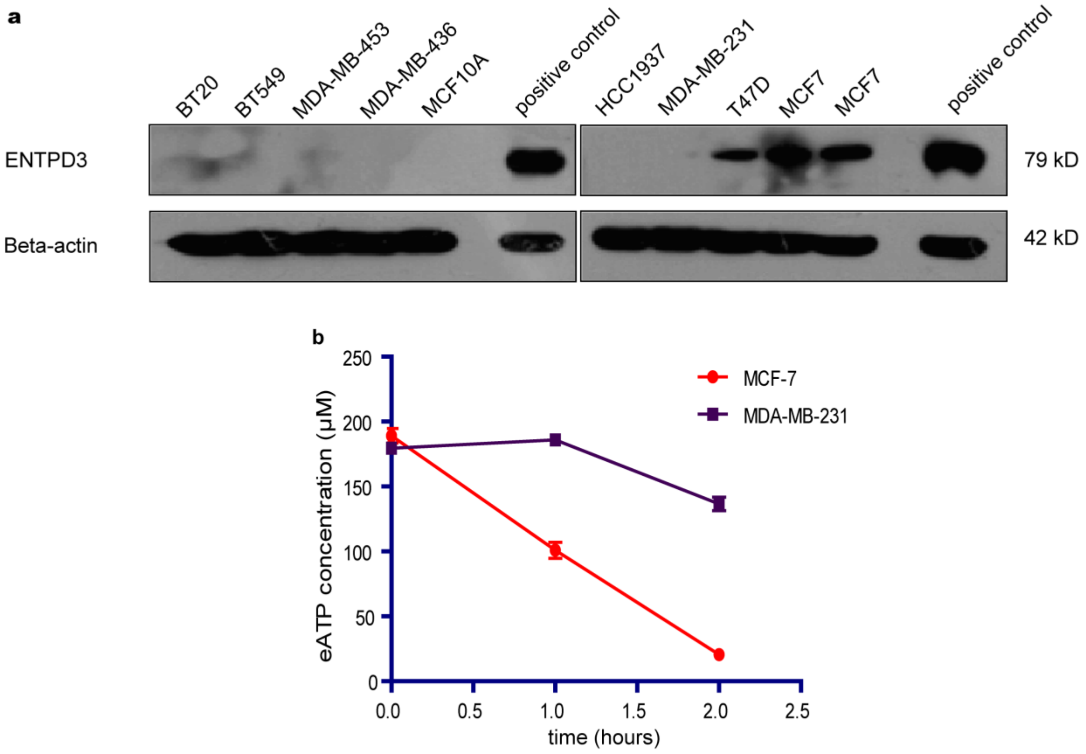


**Figure. S3: The expression of ENTPD3 and the degradation of extracellular ATP in different breast cancer cells.** (a) ENTPD3 protein level in different breast cancer cell lines. (b) Curve showing extracellular ATP hydrolysis in MCF-7 or MDA-MB-231 cells.

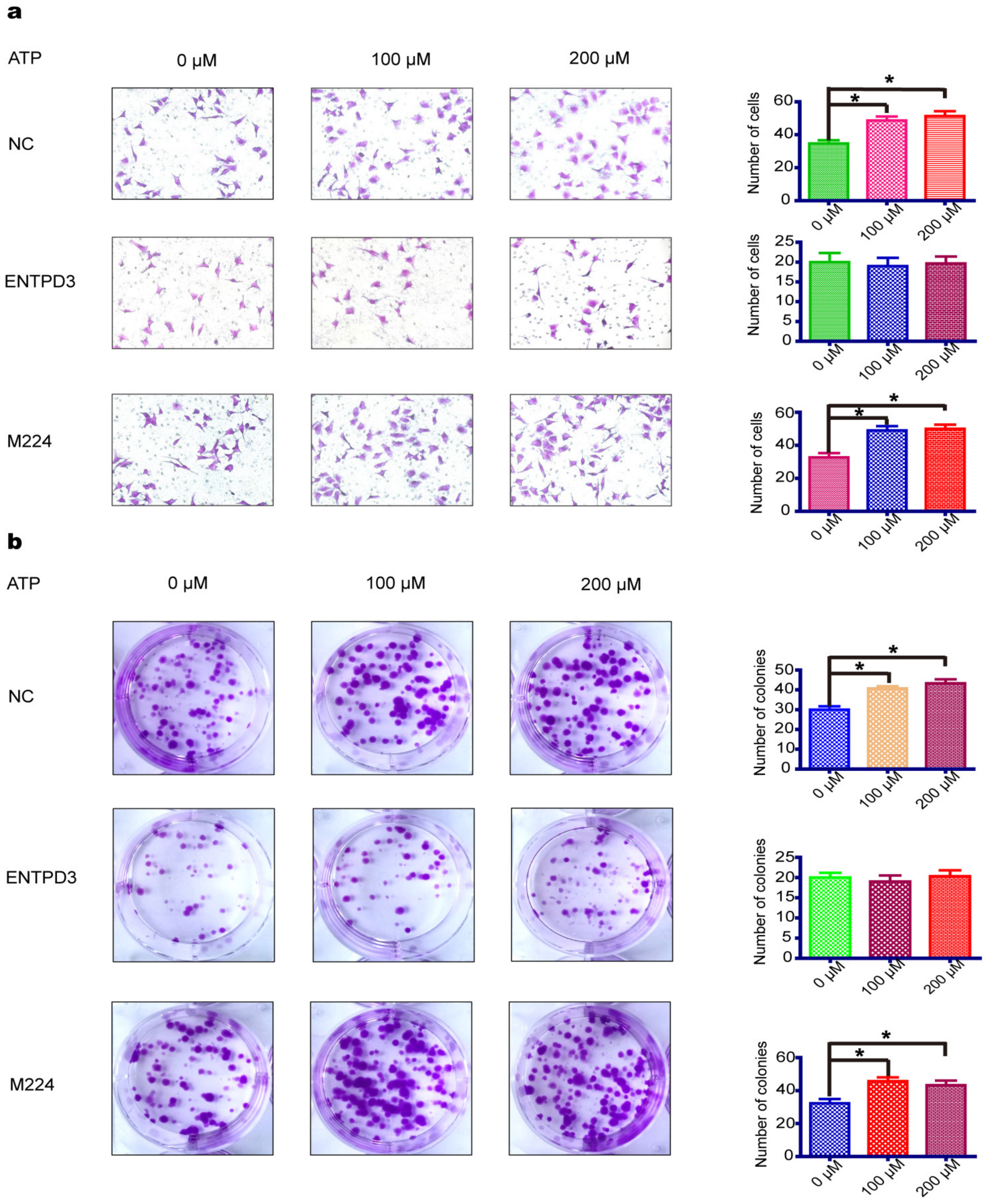


**Figure. S4: ENTPD3 reverses pro-metastasis of eATP in breast cancer.** (a) Migration of MDA-MB-231 cells treated with different doses of eATP (* $p$<0.05, $t$-test). (b) Colony formation ability of MDA-MB-231 cells treated with different

doses of eATP (* $p<0.05$, *t*-test). NC, normal control, M224, M224 mutagenesis of ENTPD3 serine 224 to alanine. Error bars, s.e.m. Repeated three times for each experiment.

| Group | Tumor | No tumor | Total | Tumor ratio |
|---|---|---|---|---|
| Control | 12 | 0 | 12 | 1.00 |
| ENTPD3 | 7 | 3 | 10 | 0.7 |
| M224 | 14 | 0 | 14 | 1.00 |

**Figure. S5: Grouping and sample size of animal experiments.**

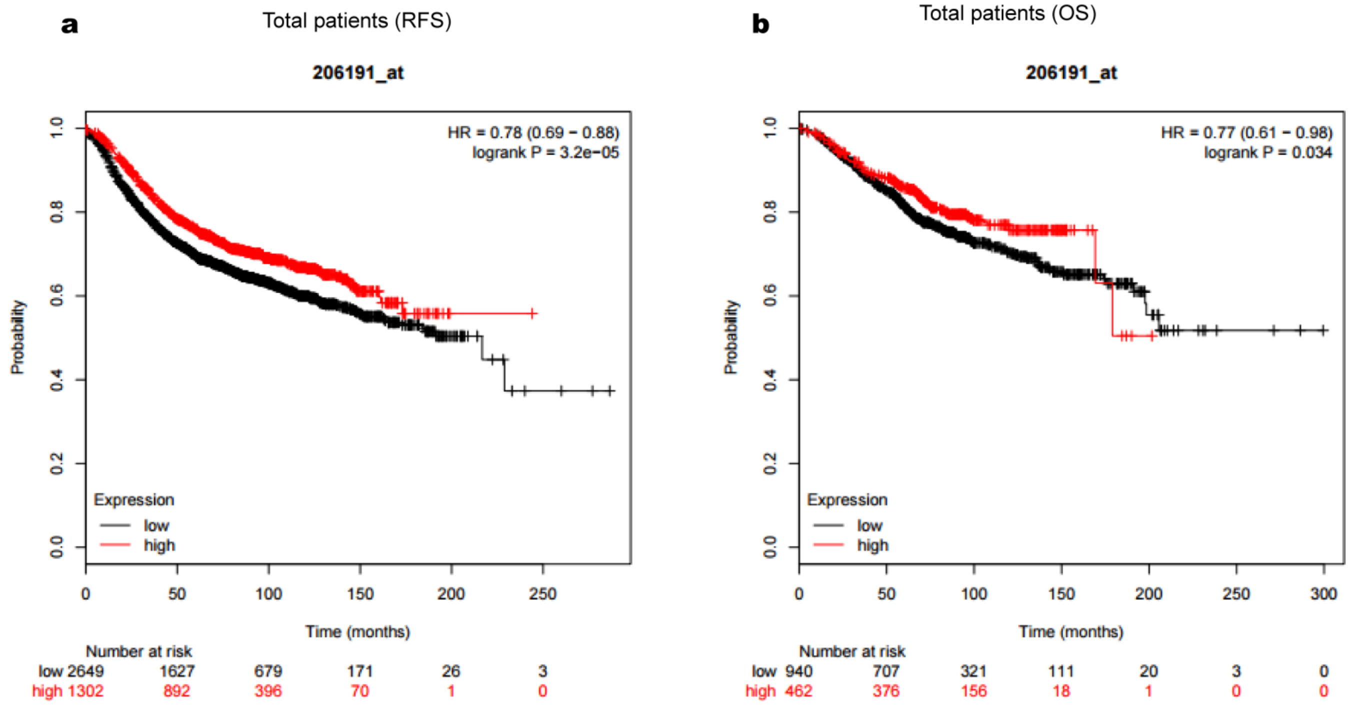


**Figure. S6: Patients with high ENTPD3 have a good outcome from the Kaplan Meier plotter database.** (a) Recurrence free survival curve in all patients. (b) Overall survival curve in all patients.
